# Good Food and Good Digestion

**Published:** 1884-02

**Authors:** 


### GOOD FOOD AND GOOD DIGESTION.

All persons who like good and well-cooked food should digest well, or they cannot properly enjoy. It takes away all the comfort of eating to know that the stomach will refuse to comfortably dispose of the luxuries presented to it. To all whose digestion is in the least degree languid, we say relieve yourself by taking Vitalized Phosphites ; it will be a permanent blessing to you, and strengthen your digestion as well as your nerves. Many hard-working persons, especially those engaged in brain work, would be saved from the fatal resort to chloral and other destructive stimulants if they would have recourse to a remedy so simple and so efficacious. This is no secret remedy ; it is used by all physicians. All who are troubled with their digestion, or with nervous weakness, go to your druggist and get a bottle of Vitalized Phosphites.—*Editor of Home Life.*

